# Development of optokinetic tracking software for objective evaluation of visual function in rodents

**DOI:** 10.1038/s41598-018-28394-x

**Published:** 2018-07-03

**Authors:** Francisco Segura, Justo Arines, Ana Sánchez-Cano, Lorena Perdices, Elvira Orduna-Hospital, Lorena Fuentes-Broto, Isabel Pinilla

**Affiliations:** 1 Department of Applied Physics, University of Zaragoza, Zaragoza, Spain; 2 Aragon Institute for Health Research (IIS Aragón), Zaragoza, Spain; 3 Department of Applied Physics, University of Santiago de Compostela, Santiago de Compostela, Spain; 4 IACS, Zaragoza, Spain; 5 Department of Physiology and Pharmacology, University of Zaragoza, Zaragoza, Spain; 6 Department of Surgery, Gynecology and Obstetrics, University of Zaragoza, Zaragoza, Spain

## Abstract

The aim of this study was to develop software that performs the optokinetic tracking assessment without the involvement of experimenters to increase the objectivity of the test. To check the effectiveness of the software, several videos were analyzed and the results were compared to those produced by two experimenters. Videos consisted of visual acuity and contrast sensitivity tests on normal animals and pigmented P23H rats (animal model of retinitis pigmentosa). Our software showed a reasonably high success rate: in approximately 78% of the cases, both the software program and the experimenters were in agreement, including the direction of rotation. The software detected 7% false positive cases, 10% false negative cases, and it was wrong in 5% of the cases. Decrease in visual function with age in pigmented P23H rats was observed from the first time interval, although minimum thresholds were found in visual parameters at advanced ages. We developed simple software based on current functions included in the Matlab image processing toolbox that was able to recognize, with a reasonably high percentage of success, the subtle head movements of the rodent produced when visual perception of the optokinetic optotype occurs.

## Introduction

Retinal neurodegenerative diseases are one of the leading causes of blindness worldwide. They include different pathologies such as age-related macular degeneration and retinitis pigmentosa (RP), and both pathologies involve retinal pigment epithelium and/or photoreceptor degeneration. RP is the most common cause of hereditary retinal degeneration^1,2^ and includes a large number of variations characterized by rod and cone progressive loss, with both functional and anatomical repercussions^3^.

Animal models are commonly used to recreate the specific features of diseases. Rodents, including both mice and rats, have been genetically manipulated to have features that resemble different retinal diseases. It is easy to maintain the rodent model and housing and testing the animals is not expensive. In both anatomical and functional testing it is possible to study the features of the disease. Usually, anatomical evaluation in animal models is performed by immunohistochemistry that employs specific antibodies or by different imaging techniques including confocal scanning laser ophthalmoscopy or optical coherence tomography. These procedures can be used to identify different cell types and their synaptic connectivity. Functional assessment is carried out using electroretinography (ERG) or optokinetic tracking systems^4,5^. ERG is one of the most widespread methods used to assess visual function in animal models. Ganzfeld ERG provides data on retinal function, however, retesting with ERG can generate cataracts or corneal opacification^6–8^. The development of other devices to test visual function, such as OptoMotry© (CerebralMechanics, Lethbride, Alberta, Canada)^9,10^, a non-invasive method for assessing optokinetic tracking of rodents *in vivo*, allows for the evaluation of photoreceptor degeneration over time as well as for testing therapeutic interventions without animal euthanasia. Although other systems based on behavioral and reinforcement tests have been used to assess vision in rodents, optokinetic tracking has characteristics that set it apart. Optokinetic tracking has a robust performance and does not require training the rodents, allowing for the quick assessment (and at earlier ages) of visual features such as visual acuity (VA) and contrast sensitivity (CS)^11–14^. However, the main disadvantage of optokinetic tracking is that it is a subjective method in which the decision about whether the animal is performing the optokinetic tracking or not is made by an experimenter.

The aim of this study was to develop software that performs the optokinetic tracking assessment without the involvement of experimenters to increase the objectivity of the test.

## Results

### Visual function of pigmented P23H rats

**Figure 1:**
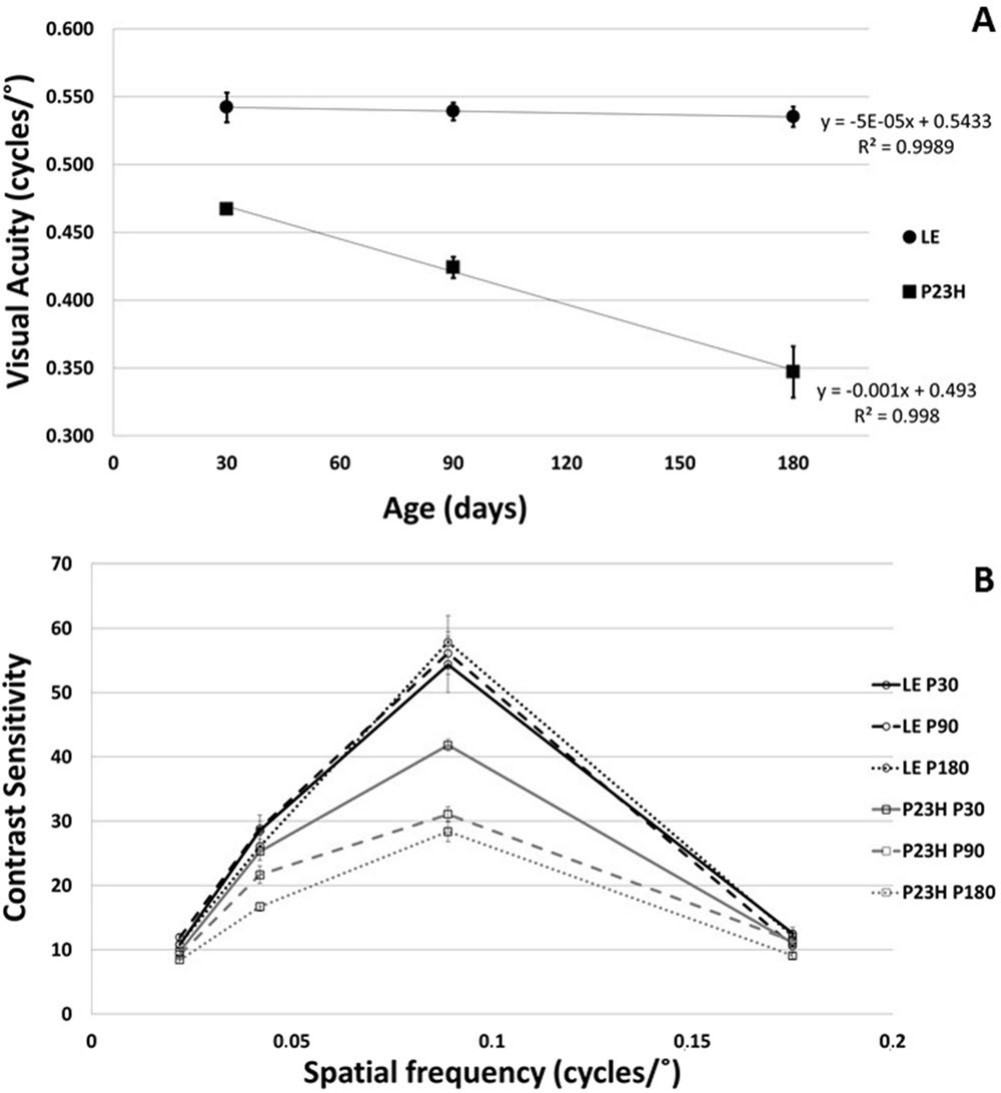
(**A**) Visual acuity of LE (circle) and P23H (square) rats as a function of age. Goodness of the fit, LE and P23H, respectively: VA = −5 × 10^−5^*age + 0.5433 (R^2^ = 0.9989) and VA = −0.001*age + 0.4930 (R^2^ = 0.9980) (**B**) Contrast sensitivity as a function of spatial frequency. Measures of the wild type control LE (circle, black line) and P23H (square, gray line) rats at P30 (continuous line), P90 (discontinuous line) and P180 (dashed line) were carried out. Each point represents the mean of 8 animals. Error bars represent the standard error of the mean.

Rats were first evaluated by an experimented observer. LE rats reached VA values (mean ± SEM) of 0.542 ± 0.011 cycles/deg at P30, 0.539 ± 0.006 cycles/deg at P90 and 0.535 ± 0.007 cycles/deg at P180. No differences with age were found. In the group of pigmented P23H rats, VA values of 0.471 ± 0.002 cycles/deg at P30, 0.428 ± 0.008 cycles/deg at P90 and 0.342 ± 0.019 cycles/deg at P180 were obtained. A progressive VA loss with age was observed (Fig. 1A).

The same trend was observed for the CS curve (Fig. 1B), with similar results in the control group and decreases with age in the pathological group for intermediate frequencies. Peaks of 54.39 ± 4.37 (P30), 56.16 ± 3.29 (P90) and 57.85 ± 4.15 (P180) in LE rats and 41.85 ± 0.89 (P30), 31.72 ± 1.16 (P90) and 28.39 ± 1.60 (P180) in pigmented P23H rats were obtained for a spatial frequency of 0.089 cycles/deg. Significant changes between species and ages have been found in all VAs and at the intermediate spatial frequencies.

### Optokinetic tracking software

Usually, one or more experimenters judge the existence or absence of optokinetic tracking in the assessment of visual function in rodents. Using motion detection software, however, could increase objectivity of the test. To check the effectiveness of the software, ten videos were analyzed. Independently the same videos were judged by two experienced operators. Each video consisted of VA or CS tests performed on the same animal, with stimuli projected at different spatial frequencies and contrasts. Five videos (VA test, CS protocol at 0.022 cycles/deg, 0.042 cycles/deg, 0.089 cycles/deg and 0.175 cycles/deg) were recorded for each type of animal (LE and pigmented P23H rat). The number of stimuli projected in each video was different depending on the tracking movements of the animals. A total of 156 events were evaluated, both by the software and the experimenters. Our software detected the projection of the stimulus in all cases, which was an important result. Each of 156 events was analyzed. A successful outcome was declared if the experimenter and software detected the optokinetic tracking (in the right direction) or if both agreed that there was no movement. Results were considered false positives if the software detected tracking but the examiner did not. If the opposite occurred, the result was considered a false negative. A result was considered wrong when the software detected optokinetic tracking in the opposite direction of rotation.

**Figure 2:**
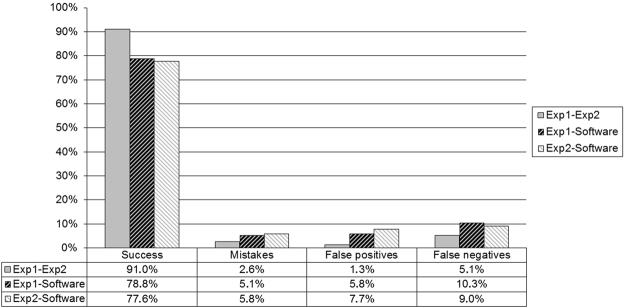
Comparison of optokinetic tracking movements detected by the software program (objective assessment) and two experimenters (subjective assessment). Gray bars represent results between experimenters; bars with black and gray stripes compare the responses of experimenter 1 (Exp1) and 2 (Exp2), respectively, with the software. Results are shown in percentages.

Movements detected subjectively by two experimenters and those obtained by the software were compared, and the results are shown in Fig. 2. Results between the two experienced researchers were compared, in 91.0% of the cases the same behavior was observed and there was a discrepancy in 9.0% of the results. In 2.6% of the experiments the tracking was detected in the opposite direction and in 6.4% of the analysis there was no agreement regarding the presence of optokinetic tracking.

**Figure 3:**
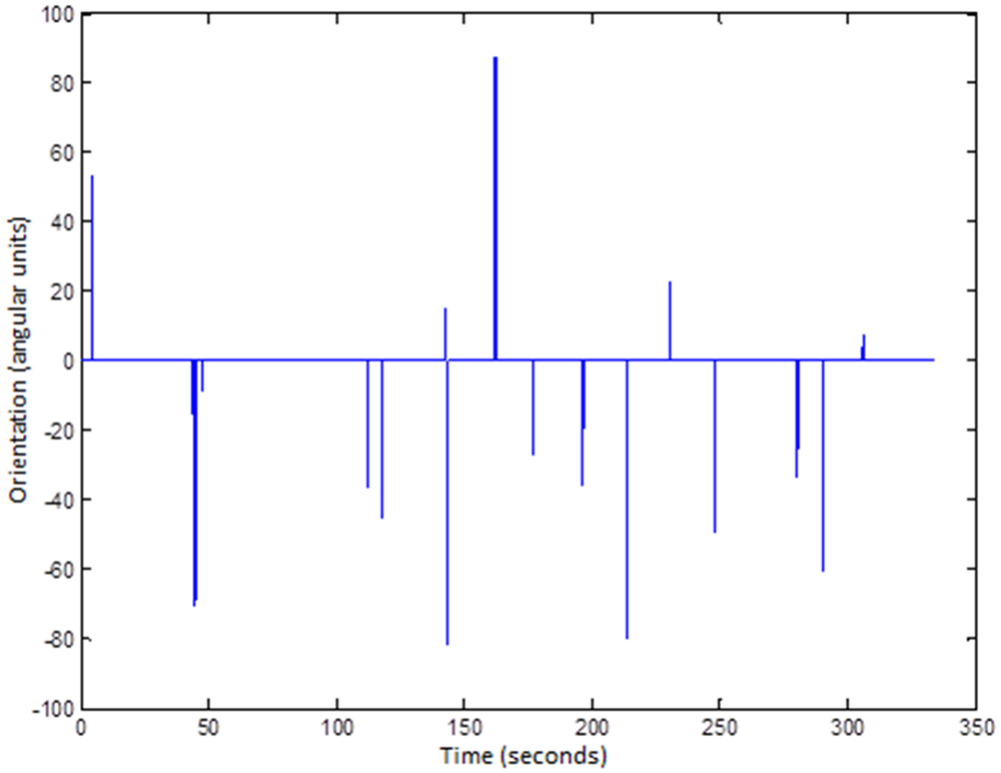
Graphical representation of the optokinetic tracking movements detected by the software in one video.

The answers of the software were compared with the results obtained by the two experimenters. In 77.6% to 78.8% of the cases, both the software program and the experimenters were in agreement, including the direction of rotation. Compared with subjective assessment, the software detected 9 and 12 false positive results (5.8% and 7.7%). Most of the false positives cases were movements detected by the software in the correct direction but not associated with optokinetic tracking. False negative results were found 16 and 14 times (10.3% and 9.0%). In all situations, the projected stimulus was close to the VA or CS thresholds, thus rodent movement was minimum. The software was wrong about the direction of rotation in 8 and 9 cases (5.1% and 5.8%), in which movements in both directions were subjectively detected. Figure 3 presents the result of the application of the software to one of the videos analyzed in the present work. Figure 3 shows the orientation of the ellipse that encloses the wake with respect to time, only for those movements that were considered as optokinetic tracking movement. So, if a value different from zero is presented, it is because a tracking movement has been identified and quantified. The orientation is only interested in terms of knowing the direction of the tracking, so its specific value is not relevant for us. Negative values informs for a counterclockwise movement, and positive value clockwise movement.

## Discussion

The OptoMotry system has been previously used to assess visual function in animal models of retinal degeneration^12,13,15,16^. However, few studies have described VA and CS thresholds of pigmented P23H rats, an animal model of RP.

Similar results to those previously published^10^ in LE rats were found. The VA values reached approximately 0.540 cycles/deg. The CS curve had a typical inverted U-shaped with slightly higher values. Both VA and CS decreased with age in the group of pigmented P23H rats, consistent with the progressive loss of photoreceptors in this neurodegenerative model. Differences between the pigmented P23H rats and the control rats were noticeable from the first temporal interval at P30, indicating that retinal degeneration appeared at early ages.

However, VA and CS values close to 0.350 cycles/deg and 30 (or 3.3%) were found at older ages (P180) in degenerative models, respectively. A late loss in cone function, compared with rods, in animals affected by a rhodopsin mutation^17,18^ could explain these findings. Thus, even P23H line 1 rats, with a faster retinal degeneration, maintained acceptable visual function at advanced ages, similarly to what occurs in humans. Other authors^19^ have concluded that the presence of photoreceptors was not necessary to obtain minimum values of VA and CS in an optokinetic tracking test, results that could also explain the existence of these thresholds at advanced ages in pigmented P23H rats.

A clear relationship between vision loss and the advance of retinal degeneration was found. In a previous paper^20^, we demonstrated that pigmented P23H line-1 at P180 showed a clearly reduced b-wave, and flicker fusion was statistically reduced. At P180, immunohistochemistry demonstrated that the inner retinal layer was formed only by two photoreceptor rows with an increased cone proportion. This photoreceptor loss with remaining cones could justify the thresholds and visual function shown in our study.

The main goal of this study was to develop automated software for optokinetic tracking assessment. As with any new technology, there is a learning curve associated with the use of the OptoMotry system. To use this method, experimenters need to be trained to distinguish between optokinetic tracking and normal movements in response to stimulation. Moreover, although visual function could be evaluated accurately and quickly in real time once the experimenter had been trained, testing methods with the OptoMotry system were as objective as possible. The automated test allowed for a completely objective assessment of tracking behavior. The improved objectivity in measurement of optokinetic tracking in rodents could be particularly useful for studying a large number of animals with retinal degenerations and assessing therapeutic treatments or genetic manipulations.

Our software showed a reasonably high success rate (∼80%) if we compare with manual detection, taking into account that this value reached 91.0% between the two experienced observers. Although other systems for objective assessment of optokinetic tracking with a higher degree of automation have been previously described^21,22^, our software achieved higher success rates. Previous studies used the location of the snout and the center of mass^21^ or the angular velocity^22^ to detect optokinetic tracking.

The work of Kretschmer *et al*.^22^ presents software based on centroid determination. The algorithm that they proposed starts by detecting the rodent by a color classification, establishing that those pixels that fulfill the color criterion are considered part of the rodent. Once they have the binary image, with the pixels that belongs to the rodent, they start computing weighting centroids. The weights depend on the position of the pixel with respect to the center of the platform that holds the rodent. From what we understood from their description, they use two centroids: (1) centroid of the complete rodent which gives an idea of where the rodent is (this centroid is close to the hind-quarters of the rodent). Once they know the centroid of the rodent they consider that the nose is the pixel farthest from the rodent centroid; (2) and the second centroid provides information of the position of the ears. It is obtained by computing the centroid inside a circle with its center in the nose. The positions of the nose and the centroid of the ears are used to calculate a vector that informs about the orientation of the nose, for posterior calculation of the rotation of the nose by comparison with successive images. From our point of view this procedure presents some drawbacks. The first one is defined from the position of the nose just by one pixel, in terms of its distance from the centroid of the rodent. We think that this option present high susceptibility to the uncertainty in the determination of the centroid and of the nose. Besides if we consider that the rodent can move the head up and down, we add more variability to the results. We found this problem when trying to use the centroid of the snout for characterizing the movement of the head. Vertical movements limit also the possibility of using correlation algorithms which in principle should be very efficient in detecting movement.

At the beginning of the study we tried different techniques. All of them were initially designed in order to detect the rotation or translation of a known shape (rodent head), as it will be the case if the rodent were not allowed to look up and down. This vertical movement is the main limitation to most of the algorithms that we tried initially. The vertical movement changes the vertical projection of the head and the change in its viewed shape. So those algorithms that rely on the constancy of the shape of the head failed. We are talking about correlation algorithms, or simple centroid based algorithms, which were our first choices. Thus, after realizing that the movement of the centroid of the snout, and correlation algorithms fail at classifying the head movement we opted for using the wake generated by the head movement on the image. This wake is characteristic as the wake of a ship on the sea (it might be difficult to determine the direction of a ship from an image, but if the wake is visible, it will tell you its direction of movement). Once we decided to use the wake of the movement for extracting information, we performed different analysis in order to determine the morphological characteristics of the wake in order to be able to classify the tracking movements. Finally the criteria for classification was determined by trial and error by comparing the classification proposed by the algorithm with the one made by an experienced observer. However posterior analysis of the criteria showed its relation with the acquisition parameters and stimulus presentation. In our case the time interval between frames was 0.0333 s. The distance from the center of the red-cross to the center of the wake is close to 50 pixels. The stimulus was presented at a speed of 12 deg/s. With these parameters in mind we can estimate that a rotation of one pixel equals 1.15 deg. With that in mind, it means that between two consecutive frames the stimulus rotates 12 deg/s * 0.03333 s = 0.39 deg. So, if we compute the wake by adding 10 frames, the time extension of the wake is 0.3 s and the movement of the stimulus 3.9 deg. Thus the minor semiaxis of the ellipse that contains the wake should be close to 3.9/1.15 = 3.4 pixels. We can add some uncertainty related to the latency of the visual system in the detection of the stimulus, and continuation of the head movement by the rodent after finishing the tracking. Thus we consider that the criteria found experimentally of minor axis of the containing ellipse equal or higher than 3 pixels and less than 8 is in accordance with the experimental results.

However, several false positives were found, indicating that further research should be conducted to improve the specificity of the test. Rarely, the software recognized two movements in opposite directions almost simultaneously. This was probably due to a rebound effect in the movement of the rodent, whom after finishing the optokinetic tracking movement, turns the head in the opposite direction slightly. At other times, the software program detected a single movement as two or more different movements all in the same direction, probably after a long tracking movement. This was a minor problem because it affected only the range of motion and not the existence of movement or the direction of rotation. We think that an easy improvement can be achieved if we correlated the direction of rotation of the stimulus with the one determined by the algorithm.

In conclusion, decreases in VA and CS with age in pigmented P23H rats were observed from the first time interval. The results confirmed the progressive loss of photoreceptors in this animal model, although minimum thresholds were found in visual parameters at advanced ages. We developed simple software based on current functions included in the Matlab image processing toolbox that was able to recognize, with a reasonably high percentage of success, the subtle head movements of the rodent produced when visual perception of the optokinetic optotype occurs. The correction of the rebound effect and the recognition of consecutive movements, as well as minimizing the detection of false positives, could improve the objective assessment of optokinetic tracking and increase the reliability of optokinetic tracking systems.

## Methods

### Animals

Software was used to assess both normal animals and animals that were manipulated to be a model of retinal disease. Pigmented P23H rats, an animal model of RP, were used in this study. These animals develop a medium-speed retinal degeneration that allows fast assessment of vision using functional tests. Rats were bred from a cross between normal pigmented Long Evans (LE) rats and transgenic albino homozygous P23H line 1. LE rats were used as the wild type controls. Eight animals from each group were studied by optomotor testing at P30, P90 and P180.

Transgenic rats were obtained from Dr. M. LaVail (University of California, San Francisco) and bred in a colony at the University of Zaragoza. Animals were housed and handled with the authorization and supervision of the Ethics Advisory Committee for Animal Experimentation from the University of Zaragoza. Procedures were performed in accordance with the ARVO Statement for the Use of Animals in Ophthalmic and Vision Research.

### Optokinetic system

**Figure 4:**
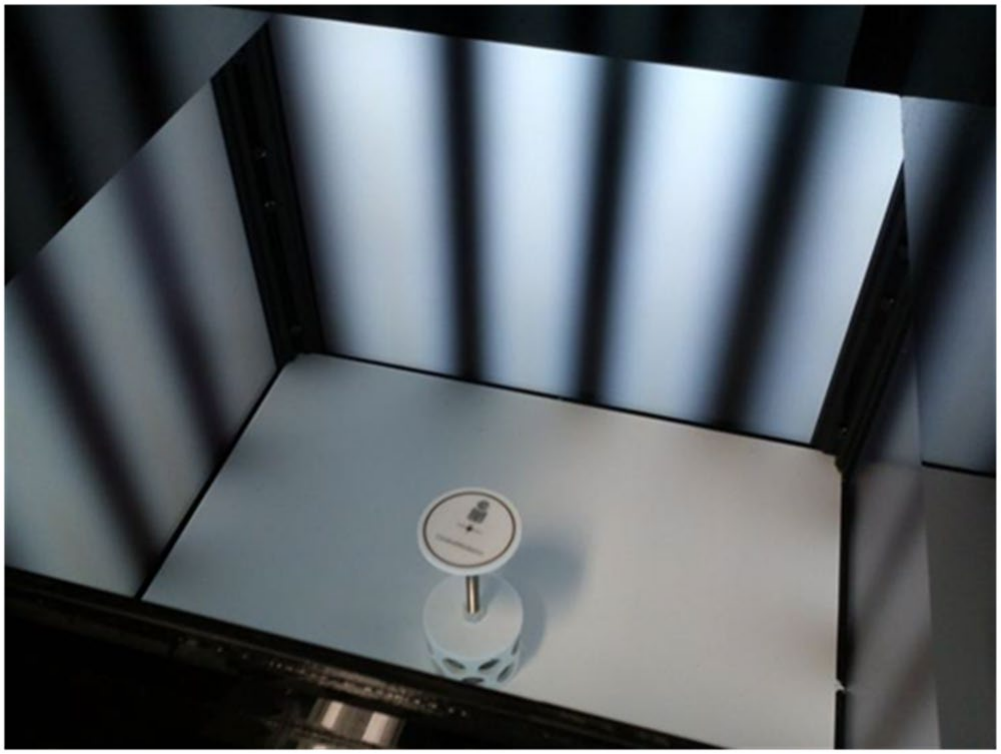
Interior view of the optokinetic system.

A 3D virtual cylinder was simulated with an OptoMotry system^9,10^ by projecting sinusoidal vertical stripes in four monitors that formed a square area. The test area consisted of a plexiglass box with rectangular openings in all side walls where the monitors were located. An elevated platform (plexiglass discs of different sizes) was situated at the center of the test area. A video camera was placed perpendicular to the platform to monitor the behavior of the animal from above. The signal was displayed on a computer connected to the system. The apparatus was located in a room with noise isolation and reduced ambient light. See Fig. 4 for details of the setup.

The virtual cylinder was projected and their characteristics were modified on the monitors by a computer program. Luminance of the screens was adjusted to equalize the intensity of the stimulus (0.20 cd/m^2^ for black stripes and 150 cd/m^2^ for white stripes). Rotation speed, direction of the cylinder and spatial frequency and contrast of the stimulus could also be controlled by the program.

To start the measurement protocol, the rodent was placed on the platform and allowed to move freely. As the rodent moved on the platform, the experimenter marked the head with a red cross superimposed on the video image. The coordinates of this cross were used to determine the center of the rotation of the cylinder in the viewing position of the animal to maintain the virtual cylinder walls at a constant distance and, thus, its spatial frequency. If a perceptible stimulus by the animal was projected, it tracked the grating with continuous and stable movements of the head and neck in the same direction as the rotation of the cylinder.

A homogeneous gray stimulus was projected at the beginning of each procedure. After the animal was placed on the platform and stopped moving, the gray stimulus was replaced by a cylinder with low spatial frequency (0.042 cycles/deg) and maximum contrast (100%) rotating at a constant speed (12 deg/s). The behavior of the animal was assessed by an experimenter for 5 seconds and the gray stimulus was restored. Thus, rodent adaptation to the stimulus was reduced. If the animal didn’t perform tracking movements or the experimenter had any doubts, the stimulus was shown a second time.

Two different threshold tests were performed to assess visual function in the rodents: VA and CS. The spatial frequency thresholds were measured by systematically increasing (using a staircase method) the spatial frequency of the grating at 100% contrast until the animals no longer tracked. Firstly, a stimulus with low spatial frequency (0.042 cycles/deg) and maximum contrast (100%) was projected. The spatial frequency was progressively increased by 0.150 cycles/deg in each step until the animal didn’t detect the stimulus. Then, system projected a stimulus which spatial frequency is the average between this value and the last frequency detected by the animal. By repeating this sequence, the spatial frequency threshold can be delimited with a method of limits. The value of this threshold was considered the maximum VA. The CS test started with a stimulus at 100% contrast that was consistently reduced (75%, 50%, 25%, 12.5%, 6.2%…) while the spatial frequency was maintained until the contrast threshold was reached, using the same staircase method than VA test. This threshold was calculated with the Michelson equation, using luminance differences of the black and white stripes projected $(\frac{{L}_{max}-{L}_{min}}{{L}_{max}+{L}_{min}})$. Subsequently, the CS value was calculated as the inverse of the threshold. A CS curve was generated by identifying the minimum contrast that generates tracking over a range of spatial frequencies (0.022 cycles/deg, 0.042 cycles/deg, 0.089 cycles/deg and 0.175 cycles/deg). VA and CS were evaluated in both directions of the rotation (clockwise and counterclockwise) that corresponded to the values obtained for each eye separately (left and right eye) because only the movement in the temporal-nasal direction generated tracking^10^.

### Description of the software

To increase the objectivity of the test, a software program for analyzing optokinetic tracking movements was developed in Matlab 2013 (MathWorks, Inc., Natick, MA, USA) and the results were compared to those produced by two experimenters using several videos of VA and CS tests. The software description is detailed below.

Several approaches were tested before developing the final tracking algorithm. The first attempts for detecting the head movements associated with rodent optokinetic tracking were based on tracking the centroid of mass of the rodent and estimating the rotation of the head. However, we found that this magnitude was biased by the body of the rodent and was not very sensitive to head movements. Small head movements associated with optokinetic tracking induced small amounts of variation to the centroid of the mass. Moreover, tremors or translations of the rodent (not head rotations) may induce similar changes in the centroid. We tested also correlation methods, but as the head of the rodent can be freely moved up and down, the shape of the head changes and the correlation provides wrong results. We therefore introduced new criteria based on detecting and following the wake of the movement of the rodent. Once we defined these criteria, we introduced different constraints to identify the head movements produced during oculomotor tracking and to eliminate those movements produced by other kinds of activities, such as tremors or the normal movements of the rodent.

Considering that we were only interested in the head movements associated with optokinetic tracking movements, we analyzed different video images to analyze the characteristics of the expected wake. We found that if the wake was large, the movements were not associated with following the visual stimuli. If the wake was small, the movements were associated with tremors. (See the following paragraphs for more details on these criteria). Besides, vertical movements of the head affected the length and width of the wake, so classification based on width and length of the equivalent ellipse seemed to be appropriate.

The algorithm developed for the objective detection of the optokinetic tracking movement consisted of three main steps: (1) detection of the wake; (2) classification of the type of wake; (3) and calculation of the rotation. Now is time for presenting the different steps of the algorithm. In appendix I we provide the complete script used for the tracking.

The first step is to load the video to Matlab2013b. Then we start an iterative algorithm in order to analyze each RGB frame (named *image(j)*) of the video. The algorithm involves several steps:

1. Detection of the center of the red-cross present in the video:
  This cross is placed in the image by the operator in order start the tracking of the head. The automatic detection is made by finding the pixel with the maximum value of the image obtained as the result of subtracting the red and green channels of the RGB *image(j)*:
  1 $$\begin{array}{c}{Aux}\_{image}={{image}{(}j{)}|}_{{Red}{channel}}\,-\,{{image}{(}j{)}|}_{{Green}{channel}}\\ {CrossCenter}={find}({Aux}{\_}{image}=={maximum}({Aux}{\_}{image}));\end{array}$$
2. Image preparation
  Before starting the analysis we convert the image to gray scale and then we select a squared region of 200 × 200 pixels around the center of the red-cross.
  2 $$\begin{array}{c}{for}\,{\rm{h}}=1:\mathrm{10}\\ {Cropped}\_{image}{(}j\,-\,h{)}={rgb2gray}{(}{Cropped}{\_}{image}{(}j-h{)}{)}{,}\\ {end}\\ {A}={Cropped}\_{image}{(}j{)}{,}\,{{A}}_{{norm}}=A{/}{maximum}{(}A{)}\\ {B}=\sum _{{\rm{h}}={\rm{1}}}^{{\rm{10}}}{Cropped}\_{image}{(}j-h{)}{;}\,{{B}}_{{norm}}=B{/}{maximum}{(}B{)}\end{array}$$
  In Fig. 5A,B we present images *A*_*norm*_ and *B*_*norm*_. In Fig. 5C we show the image resulting from the subtraction *B*_*norm*_ *−* *A*_*norm*_.

**Figure 5:**
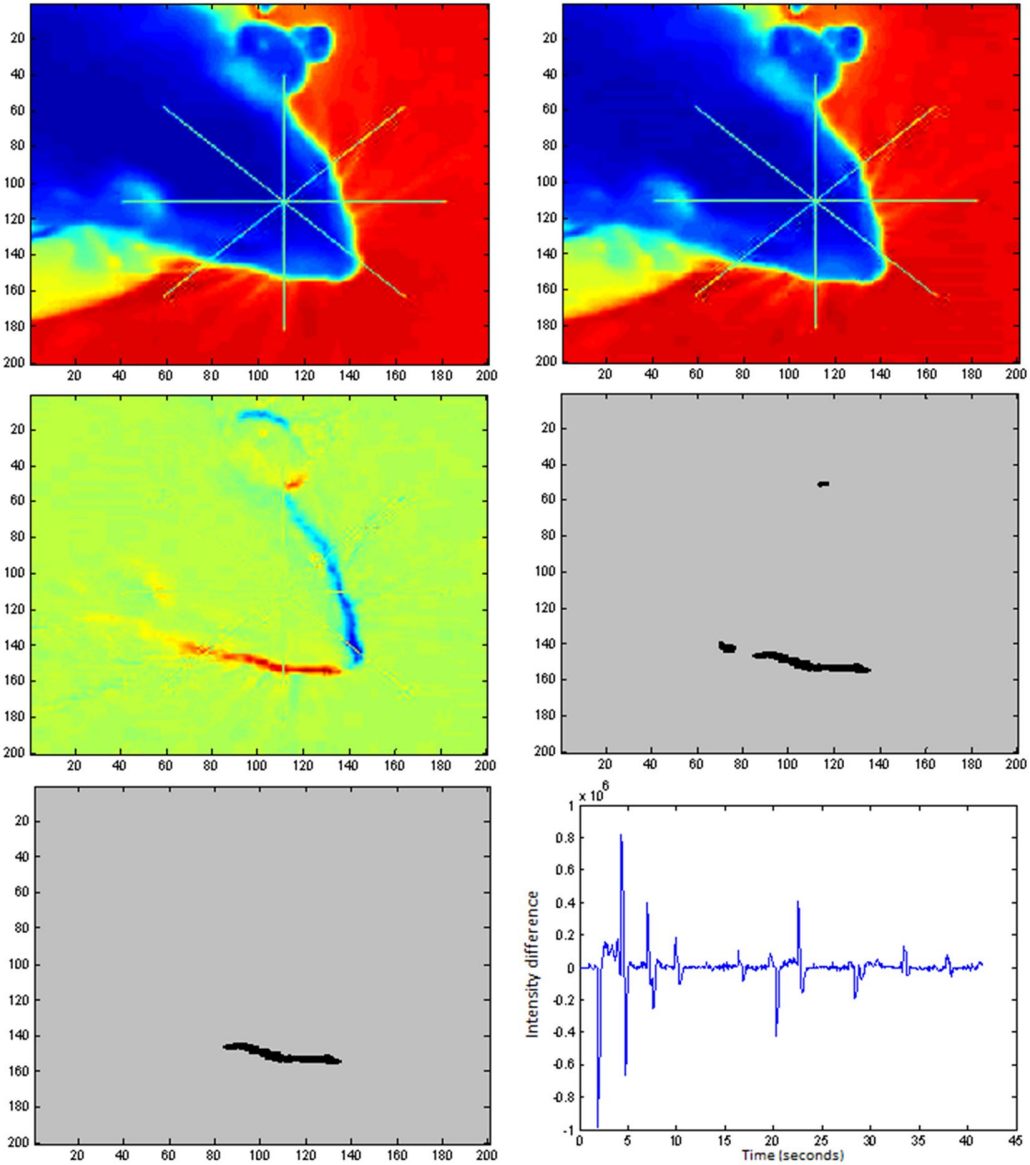
(**A**) *image(j)*. (**B**) Average of the 10 previous images to *image(j)*. (**C**) Image obtained from subtraction *B*_*norm*_ *−* *A*_*norm*_. (**D**) image of the wake with detected objects. (**E**) Single object that met all morphological requirements. (**F**) Graphic representation of the values of the total intensity of the image obtained from the subtraction *B-A* (no normalization has been applied to B or A).
3. Creation of the wake image
  For creating the image of the wake we subtract *B*_*norm*_ *−* *A*_*norm*_. Then we perform a logical operation in order to find the pixels above a threshold (which constitutes the wake).
  3 $${W}{A}{K}{E}={b}{w}{m}{o}{r}{p}{h}(({{B}}_{{n}{o}{r}{m}}-{{A}}_{{n}{o}{r}{m}}) > 0.25^{\prime} {,}{majority}^{\prime} )$$
  The matlab function, bwmorph, (‘majority’), sets a pixel to 1 if five or more pixels in its 3-by-3 neighborhood are 1’s, by doing this we smooth the boundary of the object. In Fig. 5D we show the image of the wake obtained from 5C.
4. Morphological image processing for detection of head movement
  This is the main part of the algorithm, in which the different morphological operations are performed in order to detect and classify the optokinetic tracking movement. It involves several steps.
  4.1. Find connected objects in WAKE
    *cc* = *bwconncomp(WAKE);*
  4.2. Calculate morphological properties of identified objects
         We calculate the area, major axis length and minor axis length of the objects considering them as equivalent ellipses.
            stats = regionprops(cc, ‘Area’);
            stats1 = regionprops(cc, ‘MajorAxisLength’);
            stats2 = regionprops(cc, ‘MinorAxisLength’);
  4.3. Find those objects that meet the selection criteria based on the morphological properties
         *idx* = *find([stats1*.*MajorAxisLength]* >*30 & [stats2*.*MinorAxisLength]* > = *3 & [stats2*.*MinorAxisLength]* <*8);*
  4.4. Labelling of the surviving objects
         *BW2* = *ismember(labelmatrix(cc)*, *idx);*
  4.5. Detection of excessive movement
         *We detect excesive movement in order to switch off the analysis or not*
         *ExcesiveMovement* = *find([stats2*.*MinorAxisLength]* >*15);*
         *if ExcesiveMovement* ~= 0
         switch = 0;
        else
         switch = 1;
        end
  4.6. Find connected objects in processed image BW2. If excessive movement is found, then the image is set to zero in order to remove from the image all the objects.
       *cc* = bwconncomp (BW2*switch)
  4.7. Calculate morphological properties of identified objects in the processed image cc. In this case we analyze the eccentricity of the equivalent ellipse.
       stats3 = regionprops(cc, ‘Eccentricity’);
  4.8.  Classify the object in cc in terms of their eccentricity
       idx = find([stats3.Eccentricity] > 0.99);
    In Fig. 5E we show the surviving object present in 5D that complains with all the selection criteria.
  4.9. Label the surviving objects
       BW3 = ismember(labelmatrix(cc), idx);
  4.10. Control step for verification of the survival of just one object. If more than one object survives the image is set to zero in order to remove all the objects.
      *ExcesiveObjects* = *max(max(bwlabel(BW3)))* == *1;*
      *if ExcesiveObjects* ~= 1
       switch = 0;
      else
       switch = 1;
      end
       *ProcessedImage* = BW3*switch;
5. Calculation of rotation of equivalent ellipse of surviving object
  At this point we have a binary image with just one object or zero objects. In case of one surviving object we proceed with the calculation of the orientation and centroid of the equivalent ellipse.
  5.1. Calculation of the centroid and orientation of the equivalent ellipse
    *Data* = *regionprops(ProcessedImage*, *‘Orientation’*,*‘Centroid’);*
  5.2. We use one final control element. We calculate the radial coordinate of the centroid. As we expect a rotation of the head the radial coordinate of the centroid should be constant or at least change slightly, we decided to allow a change in the radial coordinate of the centroid between two consecutive wakes less than 3 pixels.

*Radial(j)* = *sqrt((Centroid(j*, *1))*.^2 + *(Centroid(j*, *2))*.^2)

*Orientation(j)* = *Orientation*.*((Radial(j)-Radial(j - 1)) <*3);*

In Fig. 5F we show a graph with the values of the total intensity of the image obtained from the subtraction *B-A* (notice that this magnitudes has not been normalized to the maximum) on each iteration of the algorithm. Note that the software was also able to detect peaks associated with the appearance and disappearance of the visual stimuli, acting as a control switch that turned rodent motion detection on and off to limit the analysis to the intervals when the stimulus was visible. Besides, contrary to what it is expected, the separation between peaks is not constant due to the fact that the video recording was driven manually by the operator, so that the recording was stopped when the rodent were distracted, and started again when the rodent recover attention. Besides, the decreasing amplitude of the peaks is due to the reduction of the contrast of the stimulus that we used for studying CS.

**Figure 6:**
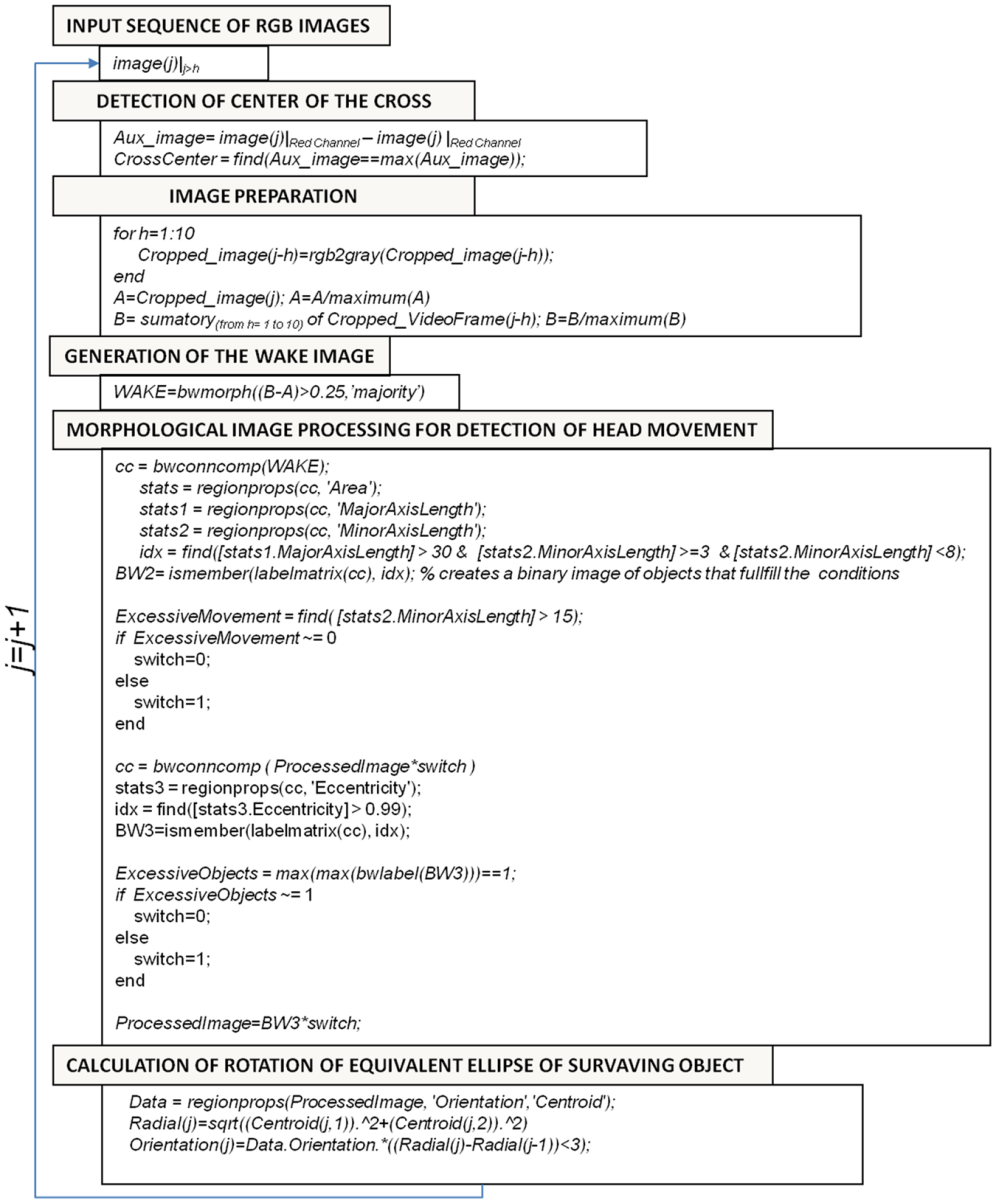
Implemented algorithm for detection of optokinetic tracking movement of rodents.

At this point we have classified *image(j)* and is the moment for come back and repeat the procedure for image j + 1 up to the end of the video. In Fig. 6 we present the complete algorithm in a more schematic way with the built in function provided by Matlab. We also provide a small video showing different steps of the algorithm during the analysis of part of one of the videos (see Movie 1) recorded in this work. Figure at the top-left presents the image obtained from the subtraction (*B*_*norm*_-*A*_*norm*_). Figure top-right presents the image of the wake. Figure, bottom-left presents the binary image obtained after application of the selection criteria based on area, and minimum and maximum axis length of the equivalent ellipse that contains the objects. Figure bottom-right present the binary image after applying all the criteria including eccentricity analysis.

Additionally, we stress that the algorithm was implemented in a very simple program in Matlab using the following built-in functions; i.e., bwconncomp, for the identification of the objects in the binary image; i.e., labelmatrixfor labeling the objects; ismember for identifying the objects that comply with the criterion; and regionprops for calculating the properties of the objects, as area, axis of the equivalent ellipse, eccentricity and centroid.

## Electronic supplementary material

Movie 1

Matlab script

## References

[1] Congdon N (2004). Causes and prevalence of visual impairment among adults in the United States. Arch Ophthalmol..

[2] Hartong DT, Berson EL, Dryja TP (2006). Retinitis pigmentosa. Lancet..

[3] Dryja, T. P. Retinitis pigmentosa and stationary night blindness. In *The Metabolic and Molecular Bases of Inherited* Disease (eds Scriver C. R., Beaudet, A. L., Sly, W. S. & Valle, D.) 5903–5933 (McGraw Hill, 2001).

[4] Berkowitz BA, Grady EM, Roberts R (2014). Confirming a prediction of the calcium hypothesis of photoreceptor aging in mice. Neurobiol Aging..

[5] van Wyk M, Schneider S, Kleinlogel S (2015). Variable phenotypic expressivity in inbred retinal degeneration mouse lines: A comparative study of C3H/HeOu and FVB/N rd1 mice. Mol Vis.

[6] Pinilla I, Lund RD, Sauvé Y (2005). Enhanced cone dysfunction in rats homozygous for the P23H rhodopsin mutation. Neurosci Lett..

[7] Cuenca N (2004). Regressive and reactive changes in the connectivity patterns of rod and cone pathways of P23H transgenic rat retina. Neuroscience..

[8] Pinilla I, Cuenca N, Martínez-Navarrete G, Lund RD, Sauvé Y (2009). Intraretinal processing following photoreceptor rescue by non-retinal cells. Vision Res..

[9] Prusky GT, Alam NM, Beekman S, Douglas RM (2004). Rapid quantification of adult and developing mouse spatial vision using a virtual optomotor system. Invest Ophthalmol Vis Sci..

[10] Douglas RM (2005). Independent visual threshold measurements in the two eyes of freely moving rats and mice using a virtual-reality optokinetic system. Vis Neurosci..

[11] Prusky GT, Alam NM, Douglas RM (2006). Enhancement of vision by monocular deprivation in adult mice. J Neurosci..

[12] Umino Y (2006). Hypoglycemia leads to age-related loss of vision. Proc Natl Acad Sci USA.

[13] Alexander JJ (2007). Restoration of cone vision in a mouse model of achromatopsia. Nat Med.

[14] McGill TJ (2012). Optomotor and immunohistochemical changes in the juvenile S334ter rat. Exp Eye Res.

[15] Zhou X, Li F, Kong L, Chodosh J, Cao W (2009). Anti-inflammatory effect of pigment epithelium-derived factor in DBA/2J mice. Mol Vis.

[16] Altimus CM (2010). Rod photoreceptors drive circadian photoentrainment across a wide range of light intensities. Nat Neurosci..

[17] Chrysostomou V, Valter K, Stone J (2009). Cone-rod dependence in the rat retina: Variation with the rate of rod damage. Invest Ophthalmol Vis Sci..

[18] Chrysostomou V, Stone J, Valter K (2009). Life history of cones in the rhodopsin-mutant p23h-3 rat: Evidence of long-term survival. Invest Ophthalmol Vis Sci..

[19] McGill TJ (2012). Discordant anatomical, electrophysiological, and visual behavioral profiles of retinal degeneration in rat models of retinal degenerative disease. Invest Ophthalmol Vis Sci..

[20] Cuenca N (2014). Correlation between SD-OCT, immunocytochemistry and functional findings in an animal model of retinal degeneration. Front Neuroanat..

[21] Benkner B, Mutter M, Ecke G, Münch TA (2013). Characterizing visual performance in mice: an objective and automated system based on the optokinetic reflex. Behav Neurosci..

[22] Kretschmer F, Kretschmer V, Kunze VP, Kretzberg J (2013). OMR-arena: automated measurement and stimulation system to determine mouse visual thresholds based on optomotor responses. PLoS One.

