# Supplementary material for: Development of optokinetic tracking software for objective evaluation of visual function in rodents
Source: Sci Rep. 2018 Jul 3;8:10009. doi: 10.1038/s41598-018-28394-x (PMC6030196; doi:10.1038/s41598-018-28394-x)
Supplement: Supplementary file 2 — Matlab script [file 41598_2018_28394_MOESM2_ESM.docx]

**Development of optokinetic tracking software for objective evaluation of visual function in rodents**

Francisco Segura, ^1,2^ Justo Arines, ^3^ Ana Sánchez-Cano, ^1,2^ Lorena Perdices, ^2,4^ Elvira Orduna-Hospital, ^2,5^ Lorena Fuentes-Broto, ^2,5^ and Isabel Pinilla ^2,4,6,*^

^1^ Department of Applied Physics, University of Zaragoza, Zaragoza, Spain

^2^ Aragon Institute for Health Research (IIS Aragón), Zaragoza, Spain

^3^ Department of Applied Physics, University of Santiago de Compostela, Santiago de Compostela, Spain

^4^ IACS, Zaragoza, Spain

^5^ Department of Physiology and Pharmacology, University of Zaragoza, Zaragoza, Spain

^6^Department of Surgery, Gynecology and Obstetrics, University of Zaragoza, Zaragoza, Spain

**APPENDIX I**

We include the Matlab script used in this work with the final algorithm:

%%%%%%%%%%%%%%%%%%%%%%%%%%%%%%%%%%%%%%%%%%%%%%%

function Orientation= FinalScriptPaperScientificReports(video)

k=0;

Orientation=0;

Rotation=0;

Centroids=[0 0];

SwitchOn=0;

SwitchSize=1;

fun = @(block_struct) ...

(mean2(block_struct.data) * ones(size(block_struct.data)))<70;

Numframes=size(video,4);

Dt=0.0333; %time lapse

Boxsize =200;

% creating of the spatial coordinates

X=1:Boxsize;

Y=X;

[Y,X]=meshgrid(Y,X);

%%%%%%%%%%%%%%%%%% MAIN LOOP %%%%%%%%%%%%%%%%%%%%%%

for j=11: Numframes

% removing of a frame of 10 pixels around the picture to prevent from % high intensity values in the border

W=(video(10:end-10,10:end-10,1,j)-video(10:end-10,10:end-10,2,j));

%detection of the center of the cross and cropping of a square of

% size (Boxsize x Boxsize) pixels

[centrox,centroy]=find(W==max(W(:)));

centroy=centroy(1);centrox=centrox(1);

R=sqrt(X.^2+Y.^2);

rect(1)=(centroy-Boxsize/2)+1;

rect(2)=(centrox-Boxsize/2)+1;

rect(3)=Boxsize;

rect(4)=Boxsize;

%%%%%%%%%%%%%%%%%%%%

for h=1:10

imagen1(:,:,h)=double(rgb2gray((video(rect(2):rect(2)+rect(4),rect(1):rect(1)+rect(3),:,j-h))));

I1(j)=max(max(imagen1(:,:,1)));

imagen1(:,:,h)=imagen1(:,:,h);

end

imagen1=sum(imagen1,3)/h;

Image1Shooting=imagen1;

imagen1=imagen1./max(imagen1(:));

% conversion of the image in grayscale

imagen2=double(rgb2gray((video(rect(2):rect(2)+rect(4),rect(1):rect(1)+rect(3),:,j))));

I2(j)=max(imagen2(:));

Image2Shooting=imagen2;

imagen2=imagen2./max(imagen2(:));

B=imagen1;

A=imagen2;

C=bwmorph((B-A)>0.25,'majority');

% detection of beginning and ending of the stimulus

sumI1(j)=sum(Image1Shooting(:))-sum(Image2Shooting(:));

if sumI1(j)<-1e6

k=j+1;

SwitchOn=0;

elseif sumI1(j)>(1e6)

SwitchOn=0;

end

if SwitchOn==0

SwitchOn=j==k;

end

if j==(k+126)

SwitchOn=0;

end

C=C.*SwitchOn; % trigger on or off

%%%%%%%%%%%%%%%%%%%%%%%%%%%%%%%%%%%%%%%%%%%%%%%%%%%%%%

% the next sentences allow for selecting the objects in the image

% in terms of their shapes

% Selection of objects with area > 15 pixels

cc = bwconncomp(C);

stats = regionprops(cc, 'Area');

stats1 = regionprops(cc, 'MajorAxisLength');

stats2 = regionprops(cc, 'MinorAxisLength');

idx = find([stats1.MajorAxisLength] > 30 & [stats2.MinorAxisLength]>=3 & [stats2.MinorAxisLength] <8);

BW2 = ismember(labelmatrix(cc), idx);

%size switch on or off

BigMovement = find( [stats2.MinorAxisLength] > 15);

if BigMovement~=0

SwitchSize=0;

else

SwitchSize=1;

end

BW3=BW2.*SwitchSize; % switching on or off BW2

% selection of objects in terms of eccentricity

cc = bwconncomp(BW3);

stats3 = regionprops(cc, 'Eccentricity');

idx = find([stats3.Eccentricity] > 0.99);

BW3 = ismember(labelmatrix(cc), idx);

% rotation switch on or off

MovementNoRotation = max(max(bwlabel(BW3)))==1;

if MovementNoRotation~=1

SwitchNoRotation=0;

else

SwitchNoRotation=1;

end

BW4=BW3.*SwitchNoRotation; % switching on or off BW3

% calculation of the orientation and centroid of surviving objects

Data = regionprops(BW4, 'Orientation','Centroid');

Orientation=cat(1, Data.Orientation)

Centroid = cat(1, Data.Centroid);

if isempty(Orientation) == 1

Orientation(j)=0;

Centroids(j,1:2)=[0 0];

Rotation(j)=0;

else

Centroids(j,1:2)=Centroid;

Radial(j)=sqrt((Centroids(j,1)).^2+(Centroids(j,2)).^2)

Orientation(j)=Orientation.*((Radial(j)-Radial(j-1))<3);

end

% display the figures and graphs

figure(1),subplot(2,2,1)

imagesc(A-B),axis image

text(10,10,num2str(Dt*j),'Color',[ 1 1 1])

figure(1),subplot(2,2,2)

imagesc(C),axis image

text(10,10,num2str(Dt*j),'Color',[ 1 1 1])

figure(1),,subplot(2,2,3)

imagesc(BW2),axis image

text(10,10,num2str(Dt*j),'Color',[ 1 1 1])

figure(1),subplot(2,2,4)

imagesc(BW4),axis image

text(10,10,num2str(Dt*j),'Color',[ 1 1 1])

figure(11),plot(Dt.*(1:j),sumI1),title(num2str(SwitchOn))

figure(12),plot(Dt.*(1:j),Orientation)

pause(0.001)

end % NumberFrames

%%%%%%%%%%%%%%%%%%%%%%%%%%%%%%%%%%%%%%%%%
